# KNOWLEDGE, ATTITUDES, AND PRACTICES TOWARD COVID-19 AMONG OLDER ADULTS IN PUERTO RICO

**DOI:** 10.1093/geroni/igac059.621

**Published:** 2022-12-20

**Authors:** Todd Becker, Thomas Buckley, Denise Burnette

**Affiliations:** University of Maryland, Baltimore, Baltimore, Maryland, United States; University of Pittsburgh, Pittsburgh, Pennsylvania, United States; Virginia Commonwealth University, Richmond, Virginia, United States

## Abstract

To better understand the dynamics of their health behaviors during the pandemic, we examined older adults’ COVID-related knowledge, attitudes, and practices (KAP). KAP theory postulates that individuals acquire knowledge about a health condition which influences their attitudes and beliefs and that these, in turn, lead to health practices. We used hierarchical regression to examine the influence of knowledge and attitudes (Step 1) on practices, controlling for health and relevant covariates (Step 2). The Step 1 association between increased knowledge and better practices (B = 0.14, p = .046) became nonsignificant in Step 2. Greater worry about contracting COVID-19 remained significant throughout (Step 2: B = 0.15, p = .043). We further explored subgroup differences within KAP measures via bivariate analyses. For instance, women had significantly higher overall knowledge (p = .013), while men had significantly better overall attitudes (p = .044). We will discuss implications of such subgroup differences for practice and policy interventions.

